# Undiagnosed Celiac Disease and Periodontal Bone Loss: A Cross-Sectional Radiological Assessment from the HUNT Study

**DOI:** 10.1155/2024/1952244

**Published:** 2024-09-02

**Authors:** Ida Haukåen Stødle, Odd Carsten Koldsland, Polina Lukina, Ina L. Andersen, Patricia Mjønes, Elin Rønne, Hedda Høvik, Eivind Ness-Jensen, Anders Verket

**Affiliations:** ^1^ Department of Periodontology Institute of Clinical Dentistry Faculty of Dentistry University of Oslo, Oslo, Norway; ^2^ HUNT Research Centre Department of Public Health and Nursing Norwegian University of Science and Technology (NTNU), Levanger, Norway; ^3^ Department of Medicine Levanger Hospital Nord-Trøndelag Hospital Trust, Levanger, Norway; ^4^ Department of Clinical and Molecular Medicine Norwegian University of Science and Technology (NTNU), Trondheim, Norway; ^5^ Department of Pathology St. Olav's Hospital Trondheim University Hospital, Trondheim, Norway; ^6^ Center for Oral Health Services and Research, Mid-Norway (TkMidt), Trondheim, Norway; ^7^ Department of Molecular Medicine and Surgery Karolinska Institutet and Karolinska University Hospital, Stockholm, Sweden

## Abstract

**Objective:**

The objective was to assess radiographic periodontal bone loss in a population with previously undiagnosed celiac disease, and to compare it to a reference group without celiac disease.

**Background:**

Periodontitis and celiac disease are chronic inflammatory diseases with possible similar features related to immune reactions and microbial dysbiosis. The relationship between these two diseases is not clear.

**Methods:**

Clinical variables, blood samples, and answers to questionnaires were collected from participants in the fourth Trøndelag Health Study (HUNT4). Celiac disease was determined based on transglutaminase 2 (TG2), immunoglobulin A (IgA), and G (IgG) in serum samples. Seropositive individuals were invited to endoscopic examination and tissue sampling. Radiographically assessed bone loss caused by periodontitis in two different levels of severity was applied as outcome, that is, ≥15% and >33% of root length. Bone loss was determined in panoramic images in participants that had attended radiographic examination in the HUNT4 Oral Health Study or in the HUNT4 Coeliac Disease Study. The association between previously undiagnosed celiac disease and radiographic bone loss was estimated by adjusted Poisson regression models.

**Results:**

Radiographic assessment was completed in 485 individuals with celiac disease determined by positive serology and in 4,727 individuals with negative serology (without celiac disease). Compared to nonceliacs, seropositive participants were less likely to present with ≥15% radiographic bone loss (prevalence ratio (PR) 0.89 (95% CI 0.84–0.96). A similar association was also observed after histopathological confirmation of celiac disease (PR 0.89 (95% CI 0.82–0.98). No association between undiagnosed celiac disease and periodontal bone loss was observed when analyses were limited to individuals with severe bone loss (>33%).

**Conclusion:**

In this study of previously undiagnosed celiac disease and periodontal bone loss, newly diagnosed celiac disease was associated with less likelihood of presenting with ≥15% radiographic bone loss compared to a nonceliac reference group.

## 1. Introduction

Celiac disease is a chronic small intestinal immune-mediated enteropathy, with varied clinical presentations as a result of autoimmunity against dietary gluten [1]. The worldwide prevalence is 1.4% [2], and the incidence seems to increase [3]. The prevalence is higher among women and in individuals with affected first-degree relatives [4, 5]. Furthermore, celiac disease is associated with other autoimmune disorders and complications such as type 1 diabetes, cardiomyopathy, Sjögren's syndrome, systemic lupus erythematosus, autoimmune hepatitis, Addison's disease, vasculitis, and osteoporosis [2, 3, 6, 7]. Diagnosis of celiac disease in adults is based on positive serology of anti-transglutaminase 2 (anti-TG2) antibodies, which are produced as a response to gluten ingestion and histological confirmation of intestinal villous atrophy [8]. The clinical presentation may vary from asymptomatic to severe symptoms caused by intestinal malabsorption. Both genetical and environmental risk factors of celiac disease have been reported [9, 10], and the composition of the gut microbiota (dysbiosis) has been hypothesized to be involved in the switch from gluten tolerance to intolerance [11].

Periodontitis is another common inflammatory disease with links to systemic health conditions. It is initiated by a dysbiotic biofilm, leading to periodontal breakdown and ultimately tooth loss if left untreated. Severe periodontitis affects about 7% of the world's population [12]. The disease is described in four stages of severity [13, 14], and the two most severe periodontal stages (III and IV), according to the 2017 classification, were reported in approximately 17% of an adult Norwegian population [15].

There is evidence to support the concept that periodontal pathogens may enter systemic circulation and that periodontitis increases the levels of systemic mediators of inflammation [16]. Periodontal disease is found to be associated with a number of other noncommunicable diseases (NCDs), such as cardiovascular disease and poorly controlled diabetes [17, 18, 19].

Periodontitis and celiac disease share common features through inflammation and damage to mucosal barrier surfaces and potentially immune system abnormality and microbial dysbiosis [1, 20, 21, 22]. A study by Cervino et al. [23] indicated that a gluten-free diet is advantageous to oral health; however, the time between intolerance onset and celiac disease diagnosis with subsequent inflammatory cessation from change of diet may suffice to elicit pathogenic effects. Hence, the time before change of diet in individuals not yet diagnosed with celiac disease is of particular interest. Further, a study has demonstrated reduced bone mineral density in patients with celiac disease [24], which has been reported to be reversed with a gluten-free diet [25, 26]. The periodontal condition in celiacs was assessed in a study which reported no association with periodontitis or attachment loss in cases with undiagnosed celiac disease [27], but only few cases were included, and larger studies were advocated.

The primary aim of this cross-sectional study was to evaluate periodontal bone loss radiographically in a population with previously undiagnosed anti-TG2 seropositivity, compared to participants without celiac disease. Secondarily, celiac disease definitions were restricted to histologically confirmed disease and to include only those that additionally exhibited symptoms as well as repeated seropositivity. The hypothesis was that radiographic bone loss in individuals with newly diagnosed celiac disease would be similar to individuals without celiac disease.

## 2. Materials and Methods

### 2.1. Study Sample: The Fourth Trøndelag Health Study (HUNT4)

**Figure 1:**
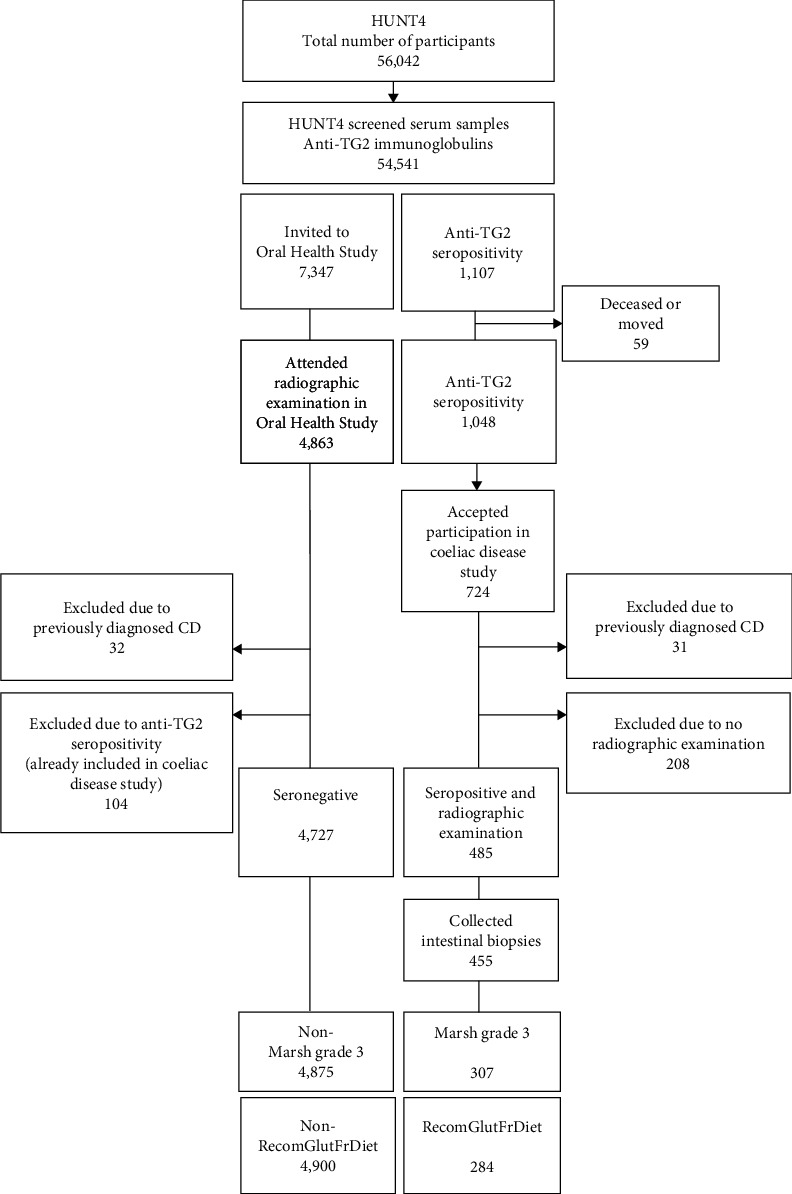
Participants with previously undiagnosed celiac disease and reference groups. Individuals with previously known celiac disease were excluded. Individuals initially included in the reference group but later diagnosed with celiac disease based on serum anti-TG2-levels were removed from the reference group and included in the celiac disease group. *Note*. CD, celiac disease; RecomGlutFrDiet, recommended to comply with a gluten-free diet; TG2, transglutaminase 2.

The investigated population was part of the Trøndelag Health Study (HUNT). HUNT is a repeated population-based study where all adult (≥20 years) inhabitants in the county of Nord-Trøndelag are invited [28, 29, 30]. Data from four surveys have been derived from 250,000 participants in total. The first study was initiated in 1984 (HUNT1). The present cross-sectional study was based on data from the fourth Trøndelag Health Study (HUNT4), which was conducted between 2017 and 2019. As for the three first HUNT studies, all residents turning 20 years within the year of participation and older were invited to participate. No exclusion criteria were applied. In all 103,800 were invited to the HUNT4 Study, and 56,042 accepted to participate [30]. A random selection of 7,347 participants, based on computer extracted social security numbers, was invited to the HUNT4 Oral Health Study. The number of invited participants to the Oral Health Study was determined by the estimated capacity at the six large field stations where the oral examinations were performed (Figure 1). Clinical measurements and blood samples, in addition to answers from questionnaires regarding systemic health, socioeconomic status, and lifestyle factors, were collected from all participants.

### 2.2. Radiographic Assessment and Data Extraction

The radiographic examination consisted of panoramic images. These were obtained with a panoramic imaging unit, Planmeca ProOne (Planmeca Oy, Helsinki, Finland), and analyzed in a room with low ambient light on a computer screen (2,560 × 1,440-pixel resolution) applying Planmeca Romexis image software, version 4.6.0.R. If reasons other than periodontitis were likely to have caused the bone loss (e.g., localized bone loss in proximity to impacted teeth or areas with alveolar bone atrophy and multiple missing teeth where tooth loss could be attributed to reasons other than periodontitis), it was disregarded, and another tooth in the dentition was selected for the severity assessment. Radiographic annotation was determined from measurements in the radiographs, which is considered reliable for periodontal bone loss assessment [31]. The distance from the cementoenamel junction (CEJ) to the top of the alveolar crest (AC) where the periodontal ligament (PDL) presented normal width [32] and the distance from the CEJ to the radiographic apex were measured in millimeter. This was carried out in the entire dentition to calculate percentage of bone loss for each tooth to identify the most severely affected mesial or distal site, which was considered for further analysis. Third molars were excluded.

### 2.3. Celiac Disease Examination

For detection of celiac disease, all serum samples collected in HUNT4 were analyzed for presence of anti-TG2 IgA and IgG antibodies. This was performed at the Department of Biochemistry, Oslo University Hospital, with a dual anti-TG2 IgA and IgG serological assay. Seropositivity was defined as anti-TG2 IgA ≥ 0.7 mg/L or anti-TG2 IgG ≥ 1.0 mg/L [33]. Individuals with positive serology were invited to participate in the HUNT4 Coeliac Disease Study and to a clinical assessment, including upper endoscopy with small intestinal tissue sampling. Tissue samples were collected from the third part of the duodenum (D3) and from the duodenal bulb (D1) by two trained gastroenterologists (ENJ and ILA) at Levanger Hospital, Nord-Trøndelag Hospital Trust. A diagnosis of celiac disease was confirmed by intraepithelial lymphocytosis, crypt hyperplasia, and villous atrophy, corresponding to Marsh [34] grades 3a, 3b, or 3c in any of the biopsies. The specimens were assessed and graded by expert pathologists (PM and ER). To attain recommendation of a gluten-free diet, participants were also required to exhibit symptoms, be seropositive on repeated testing, and without other possible explanations of the intestinal alterations. Participants in HUNT4 with previously diagnosed celiac disease were identified through the Norwegian Patient Register and through hospital records and excluded from the analyses. A panoramic radiographic image was offered to all participants that attended endoscopy, if not already performed as part of the HUNT4 Oral Health Study. Participants with seropositivity were recruited from the total study population, regardless of participation in the Oral Health Study. Seropositive participants who had attended the Oral Health Study were excluded from the latter and included in the Coeliac Disease Study (Figure 1).

### 2.4. Outcome and Exposure of Interest

The outcome was radiographically assessed bone loss caused by periodontitis. Bone loss was divided into categories (<15%, ≥15%–33%, and >33%) corresponding to the stages of severity according to the 2017 classification [14, 35, 36]. Two different levels of bone loss severity were applied as outcome, that is, ≥15% and >33%. The exposure was celiac disease defined in three ways based on: (1) seropositivity (TG2 IgA ≥ 0.7 mg/L or IgG ≥ 1.0 mg/L); (2) additional histological confirmation of Marsh grade 3a, 3b, or 3c; and (3) additional recommendation of a gluten-free diet based on the clinical assessment and repeated seropositivity.

### 2.5. Confounders

Confounders were selected based on variables that potentially influence periodontal bone loss and included, sex, age (continuous), HbA1c-level (continuous), smoking (never, former, and current), years of education (9–10 years, 11–13 years, and college/university), and total gross household income (≤70,000 and >70,000 EUR). Information about sociodemographic and lifestyle factors was assessed via self-reported questionnaires. Blood samples were drawn and analyzed for hemoglobin A1c (HbA1c) in nonfasting serum or whole blood. Two separate enzymatic analyses, HbA1c and total hemoglobin (THb), were used to calculate the percentage of glycogen hemoglobin according to the National Glycohemoglobin Standardization Program (NGSP) and the hemoglobin fraction according to the International Federation of Clinical Chemistry and Laboratory Medicine (IFCC) of HbA1c (Reagent kit; 4P52-21 Hemoglobin A1c, Multigent, Abbot Laboratories, USA). Missing responses in the questionnaires were reported.

### 2.6. Calibration

Examination of radiographs was performed by three specialists in periodontology (IHS, AV, and OCK). Interinvestigator calibration was performed on radiographs from 70 participants. Each of the investigators evaluated the 70 panoramic images separately and registered percent of bone loss of the most severely affected tooth in the dentition.

### 2.7. Statistics

Descriptive statistics are presented as mean and standard deviation for continuous variables and percentages with 95% confidence intervals (CI) for categorical variables. One-way ANOVA was used to assess significant differences across group means for continuous variables, and Pearson chi-square test was used for categorical variables. For the main analysis, multivariable Poisson regression with robust variance was used to estimate the prevalence ratios (PRs) with 95% CI of the associations between celiac disease and radiographic bone loss in categories (≥15 and >33%). A linear multivariable regression model with bone loss on a continuous scale was applied as a sensitivity analysis despite the expectation of periodontitis occurrence as nonlinear. This was performed to examine the effect of celiac disease as bone loss progresses from no to severe bone loss. Further sensitivity analyses were performed of never-smokers only and by propensity score matching, assessing treatment effects on a binary outcome calculated with logistic regression and 1 : 4 matching using the nearest neighbor algorithm. The latter was performed to evaluate possible covariate differences between the celiac and nonceliac groups. All models were adjusted for potential confounders using a stepwise approach. The selection of confounders was based on variables considered to be associated with the outcome. Missing variables were included as categories in the initial comparison of population characteristics between groups (Table 1). In the regression analyses, only complete cases were included.

Interrater reliability for radiographic bone loss assessment (percentage of root length) was calculated using intraclass correlation coefficient (ICC) two-way mixed effects, assessing consistency. The ICC (95%) of the radiographic assessment of percent of periodontal bone loss in the 70 calibration cases was ICC 0.95 (95% CI 0.981–0.964). The statistical analyses were performed using Stata/MP 16.0 (Stata Corp., TX, USA).

### 2.8. Ethics

The HUNT4 Study was approved by the Norwegian Data Protection Authority. The current study was performed in accordance with relevant guidelines and regulations and was evaluated and approved by the Norwegian Regional Committees for Medical and Health Research Ethics (2016/1879/REK, 2020/10417/REK, and 2021/330940/REK). Informed consent was obtained from all participants and/or their legal guardians. The paper was prepared following the STROBE guidelines.

## 3. Results

Of the 54,541 HUNT4 participants with serum samples, 1,107 presented with positive serology (i.e., based on levels of anti-TG2, immunoglobulin A (IgA), and G (IgG)) and were eligible for invitation to the HUNT4 Coeliac Disease Study. Of these, 724 accepted to attend clinical examination with endoscopy, biopsies, and an oral radiographic examination, if not already performed. Due to previously diagnosed celiac disease, 31 participants were excluded. Another 208 participants were excluded due to missing oral radiographs. Oral radiographic examination was performed in 485 seropositive participants without previously known celiac disease. Biopsies were collected from 455 participants, of which 307 were registered with Marsh grade 3a, 3b, or 3c. Of these, 284 were further recommended to follow a gluten-free diet (Figure 1). All participants that had attended the HUNT4 Oral Health Study and who had received a radiographic examination (*n* = 4,863) were considered eligible as controls and included in the reference group. Due to previous diagnosis of celiac disease, 32 individuals in the reference group were excluded. Furthermore, 104 participants from the Oral Health Study were among those with anti-TG2 seropositivity without former celiac disease diagnosis and were thereby moved from the reference group and included in the celiac disease group. This resulted in a reference group of 4,727 participants. When the exposures were limited to participants with histological confirmation of Marsh grade 3 and further participants who fulfilled requirements for recommendation of a gluten-free diet, the reference groups increased accordingly to 4,875 and 4,900 participants.

Characteristics of participants with previously undiagnosed disease (based on anti-TG2 seropositivity) and nonceliac references are presented in Table 1. The mean percentage of bone loss was 15.9 (14.7) and 18.3 (15.4) among celiacs and nonceliacs, respectively. Among participants with celiac disease based on positive serology, 54.8% (50.1–59.4) was registered with radiographic bone loss ≥15%, while the corresponding number was 59.3% (57.9–60.7) in the reference group.

### 3.1. Association with Celiac disease

In the adjusted model, individuals with celiac disease (anti-TG2 seropositivity) presented with lower likelihood of having radiographic bone loss ≥15% compared to the nonceliacs (PR 0.89 (95% CI 0.84–0.96); Table 2). This was also observed for individuals with Marsh grade 3 (PR 0.89 (95% CI 0.82–0.98); Table 3) and for individuals who were recommended to follow a gluten-free diet (PR 0.91 (95% CI 0.83–1.00)) (Table 4), as compared to the respective reference groups. No significant associations between newly diagnosed celiac disease and radiographic bone loss were observed when bone loss was limited to severe cases (>33%). Analysis of radiographic bone loss as a continuous measure and celiac disease based on positive serology (coef. −2.97, 95% CI −4.00, −1.95), Marsh grade (coef. −2.79, 95% CI −4.06, −1.52), and recommendation of gluten-free diet (coef. −2.62, 95% CI −3.94, −1.31) confirmed the findings.

### 3.2. Sensitivity Analyses

The association between celiac disease and radiographic bone loss ≥15% or >33% did not substantially change when analyzing never-smokers only. Further agreement with the main analyses was observed from the propensity score matching. The coefficients of the associations between positive serology and ≥15% bone loss, Marsh 3 graded biopsies and ≥15% bone loss, and having been recommended to comply with a gluten-free diet and ≥15% bone loss were −0.08 (95% CI −0.11 and −0.04), −0.05 (95% CI −0.09 and −0.01), and −0.05 (95% CI −0.11 and 0.01), respectively. The standardized differences and variance ratios between covariate group means demonstrated good matching across groups.

## 4. Discussion

In this cross-sectional study, the proportion of participants with ≥15% bone loss was significantly lower among newly diagnosed celiacs, as compared to the reference group. No difference was observed when the analyses were limited to severe cases of bone loss (>33%).

The present findings are in agreement with the results of an NHANES study [27]. The study reported an association between previously undiagnosed celiac disease and lower mean probing depth (PD) as compared to PD in nonceliacs. No statistically significant association was reported between celiac disease and periodontitis according to Centers for Disease Control and Prevention/American Academy of Periodontology(CDC/AAP) definition [37] or mean clinical attachment level (CAL), although the odds ratio of the association with periodontitis was lower in undiagnosed celiacs than in nonceliacs, and correspondingly, the mean CAL was lower in celiacs than in the counterpart without celiac disease. The low sample size (*n* = 34) may have inflicted on these observations.

One may speculate if increased awareness and behavioral changes toward a healthier diet may play a role in the protective effect of celiac disease against bone loss, if one considers cases with known genetical inclination to celiac disease [38]. Another speculation may be the composition of the oral microbiome in celiacs, which is found to be different to that of individuals without celiac disease. Tian et al. [39] reported higher levels of *Lactobacillus* species in celiacs compared to nonceliacs. *Lactobacilli* are oral commensals, not among the bacterial complexes known to be associated with periodontitis. Moreover, the healthy controls of that study [39], that is, individuals without celiac disease, harbored higher levels of bacterial species which were compatible with periodontitis. *Lactobacilli* species have previously been demonstrated to suppress the growth of periodontal pathogens [40]. Likewise, bacterial antagonism against periodontal pathogens in nonperiodontitis patients has been shown in an in vitro study by van Essche et al. [41]. Both of these studies support the notion that the microbiome in individuals with celiac disease may be related to the negative association with periodontal bone loss observed in the present investigation. Importantly, these studies report microbiology and periodontitis, while the present study report bone loss, and caution should be taken in inferring the results.

The differences in bone loss observed between the two groups may be related to background characteristics. Individuals with an interest in good health are more prone to accept participation in research than their counterparts [30], and selection bias toward the healthier is likely [42]. It is also a possibility that the long travel distance to Levanger Hospital for enterological examination and Levanger Dental Clinic for oral radiographic examination have reduced the number of included participants from more distant municipalities to the Coeliac Disease Study. The data collection in the Oral Health Study was performed locally in the respective municipalities and may therefore represent a broader section of the Nord-Trøndelag county. Among the anti-TG2 seropositive participants, more individuals had education on college or university level among those who attended the radiographic examination as compared to those who did not. No other differences with respect to background characteristics were seen.

The sensitivity analyses of the present study were in concordance with the main analyses. The findings were also consistent through the different levels of celiac disease criteria (i.e., Marsh grade 3 and recommended gluten-free diet). The lack of association with severe cases of bone loss may partly be explained by the lower number of participants with bone loss exceeding 33% among the cases with newly diagnosed disease, which comprised only 60 individuals with anti-TG2 seropositivity and 33 who were registered with Marsh grade 3.

The participants without celiac disease in the present study comprised more than 4,000 individuals. This large number of participants is considered a strength with regard to the construction of a well-balanced and valid basis of comparison. Further strengths of the study included serum samples of a large number of individuals (*n* = 54,541) for screening of TG2 antibodies, evaluation of tissue samples by expert pathologists, and a final clinical assessment by gastroenterologists. Further, the annotation of radiographs was performed by calibrated specialists in periodontology.

There are also weaknesses to consider. A part of the participants in the HUNT4 Coeliac Disease Study was not evaluated radiographically (*n* = 208), which limited the inclusion of cases with newly diagnosed disease. Bias related to participants without radiographic evaluation cannot be ruled out. No clinical periodontal examination was performed, which in turn renders inferior estimates of periodontal conditions as compared to a comprehensive clinical assessment including CAL, periodontal probing depth (PPD), and furcation involvement. Further, bleeding on probing was not assessed to evaluate periodontal inflammation, which would be of interest. Lack of clinical assessment is considered the main limitation of the present study. Complexity factors assessable in panoramic radiographic images, such as furcation defects, tooth loss, and intrabony defects, were not included in the regression analyses, and radiographic bone loss thresholds were applied as outcome instead of periodontal stages. The thresholds of bone loss correspond to the disease severity categories of the 2017 classification. However, assessment of periodontal bone loss may have included bone loss caused by other reasons than periodontitis, thereby leading to an overestimation of periodontal tissue loss. Further, the least severe cases of periodontitis are likely to exhibit attachment levels in the intersection between normal supracrestal tissue attachment and incipient attachment loss, leading to misclassification of periodontitis cases. Importantly, this possibility will not entail differences between celiac and nonceliac participants. Finally, some of the included covariates in the statistical estimates were self-reported, and bias is possible. The PRs of the associations between bone loss and celiac disease were in the order of 0.8–0.9. Although statistically significant, the differences were small, and the clinical relevance of the findings is uncertain and perhaps negligible.

The present population is representative of Norwegian small town and countryside regions [30]. Norway is characterized by readily available health care services and resembles other Scandinavian countries. This implies that good general and dental health are common, and care should be taken in extrapolation to populations with less developed welfare services and lower socioeconomic status.

The association between celiac disease and periodontitis has not been extensively investigated previously. The findings of the present study need confirmation by additional observational or ideally prospective studies, which may more thoroughly control possible confounding.

## 5. Conclusion

The present cross-sectional study of untreated celiac disease and periodontal bone loss demonstrated a decreased likelihood of bone loss ≥ 15% in individuals with newly diagnosed celiac disease compared to a reference group, thereby rejecting the hypothesis. No difference was observed for bone loss > 33%. The present study reports only radiographical findings, and caution should be taken to avoid extrapolation to clinically assessed periodontitis.

## Figures and Tables

**Table 1:** Characteristics of study participants.

Characteristics	Reference group without celiac disease (*n* = 4,727)	Anti-TG2 seropositivity previously undiagnosed (*n* = 485)	*p*-Value
Age in years, mean (SD)	51.5 (16.4)	53.0 (15.6)	0.054
Sex, % (95% Cl)			
Men	44.1 (42.7–45.6)	47.8 (43.2–52.5)	0.316
Women	55.9 (54.4–57.3)	52.2 (47.5–56.8)	—
HbA1c mmol/mol, mean (SD)	34.0 (5.9)	34.2 (5.7)	0.621
Bone loss, continuous %, mean (SD)	18.3 (15.4)	15.9 (14.7)	0.001
Bone loss, categories, % (95% CI)			
<15%	40.7 (39.3–42.1)	45.2 (40.6–49.9)	0.153
≥15%–33%	45.1 (43.7–46.5)	42.4 (37.8–47.0)	—
>33%	14.3 (13.3–15.3)	12.5 (9.6–15.8)	—
Number of teeth, mean (SD)	25.6 (3.8)	25.7 (3.3)	0.549
Smoking, % (95% CI)			
Never	58.0 (56.6–59.4)	62.2 (57.6–66.7)	0.094
Former	35.1 (33.7–36.5)	33.4 (29.1–37.9)	—
Current	6.3 (5.7–7.1)	3.7 (2.2–5.9)	—
Missing	0.6 (0.4–0.8)	0.7 (0.1–1.9)	—
Education, % (95% CI)			
9–10 years	7.0 (6.3–7.7)	5.2 (3.4–7.7)	0.438
11–13 years	47.1 (45.7–48.6)	47.6 (42.9–52.3)	—
College/university	45.3 (43.9–46.7)	46.3 (41.6–51.0)	—
Missing	0.6 (0.4–0.8)	0.9 (0.2–2.2)	—
Income, total gross household in euros, % (95% CI)			
<70,000	53.6 (52.2–55.0)	51.1 (46.4–55.8)	0.149
>70,000	44.3 (42.9–45.7)	47.8 (43.2–52.8)	—
Missing	2.1 (1.7–2.6)	1.1 (0.4–2.5)	—

*Abbreviations*. CI, confidence interval; HbA1c, glycated hemoglobin; SD, standard deviation; TG2, transglutaminase 2. Data are presented as mean (SD) or percentage (95% CI). One-way ANOVA was used to assess significant differences across group means for continuous variables, and Pearson chi-square test was used for categorical variables.

**Table 2:** Association between previously undiagnosed celiac disease determined by antitissue transglutaminase 2 (TG2) IgA and IgG antibodies and radiographic bone loss by Poisson regression analysis.

Bone loss	No. of observations	Adjusted PR (95% CI)
Bone loss ≥15%		
TG2 seropositivity		
Model 1	5,212	0.92 (0.85–1.00)
Model 2	5,131	0.90 (0.84–0.96)
Model 3	5,033	0.89 (0.84–0.96)
Bone loss >33%		
TG2 seropositivity		
Model 1	5,212	0.85 (0.64–1.13)
Model 2	5,131	0.82 (0.60–1.12)
Model 3	5,033	0.82 (0.59–1.12)

*Note*. Reference: No TG2 seropositivity. Model 1: Crude. Model 2: Adjusted for sex; age (by 1-year increase); HbA1c (by one mmol/mol increase); and smoking (never, former, and current). Model 3: Model 2 + education (9–10 years, 11–13 years, and college) and income, tot. household in euro (≤70,000 and >70,000).

**Table 3:** Association between previously undiagnosed celiac disease determined by graded tissue samples and radiographic bone loss by Poisson regression analysis.

Bone loss	No. of observations	Adjusted PR (95% CI)
Bone loss ≥15%		
Marsh grade 3		
Model 1	5,182	0.87 (0.77–0.97)
Model 2	5,101	0.91 (0.83–0.99)
Model 3	5,004	0.89 (0.82–0.98)
Bone loss >33%		
Marsh grade 3		
Model 1	5,182	0.75 (0.54–1.05)
Model 2	5,101	0.89 (0.66–1.20)
Model 3	5,004	0.90 (0.67–1.22)

*Note*. Reference: No TG2 seropositivity or seropositivity but without tissue sample alterations corresponding to Marsh grade 3. Model 1: Crude. Model 2: Adjusted for sex; age (by 1-year increase); HbA1c (by one mmol/mol increase); and smoking (never, former, and current). Model 3: Model 2 + education (9–10 years, 11–13 years, and college) and income, tot. household in euro (≤70,000; >70,000).

**Table 4:** Association between previously undiagnosed celiac disease determined by a recommendation to attain a gluten-free diet and radiographic bone loss by Poisson regression analysis.

Bone loss	No. of observations	Adjusted PR (95% CI)
Bone loss ≥15%		
Recommended gluten free diet		
Model 1	5,184	0.87 (0.77–0.97)
Model 2	5,103	0.92 (0.84–1.01)
Model 3	5,006	0.91 (0.83–1.00)
Bone loss >33%		
Recommended gluten-free diet		
Model 1	5,184	0.76 (0.54–1.07)
Model 2	5,103	0.93 (0.68–1.27)
Model 3	5,006	0.95 (0.69–1.29)

*Note*. Reference: No TG2 seropositivity or seropositivity but without recommendation to attain a gluten-free diet. Model 1: crude. Model 2: Adjusted for sex; age (by 1-year increase); HbA1c (by 1-mmol/mol increase); smoking (never, former, and current). Model 3: Model 2 + education (9–10 years, 11–13 years, and college); income, tot. household in euro (≤70,000; >70,000).

## Data Availability

The data are stored in HUNT databank and biological material in HUNT biobank. HUNT Research Centre has permission from the Norwegian Data Inspectorate to store and handle these data. The key identification in the database is the personal identification number given to all Norwegians at birth or immigration, while de-identified data are sent to researchers upon approval of a research protocol by the Regional Ethical Committee and HUNT Research Centre. To protect participants' privacy, HUNT Research Centre aims to limit storage of data outside HUNT databank and cannot deposit data in open repositories. HUNT databank holds precise information on all data exported to different projects and can reproduce these on request. There are no restrictions regarding data export given approval of applications to HUNT Research Centre. Provided approval from HUNT Research Centre, sharing of data from the present investigation will be supported by the corresponding author upon reasonable request. For more information, see www.ntnu.edu/hunt/data. Inquiries regarding access to data are directed to kontakt@hunt.ntnu.nohttps://biobankregisteret.no/#/biobankDetails/4094, project 2020/26788.
